# Hooking recreational fishers on sustainable fishing: Consistent psycho-social determinants raise potential for broad scale interventions

**DOI:** 10.1007/s13280-025-02227-4

**Published:** 2025-08-21

**Authors:** Matthew Navarro, Tracey Mahony, Diane Jarvis, Natalie Stoeckl, Francisco Gelves-Gomez, Vanessa M. Adams

**Affiliations:** 1 UWA School of Biological Sciences, UWA Centre for Environmental Economics and Policy and UWA Oceans Institute, The University of Western Australia, 35 Stirling Highway, Crawley, Perth, WA 6009 Australia; 2 College of Business, Law & Governance, James Cook University, Bebegu Yumba Campus, 1 James Cook Dr, Douglas, Townsville, QLD 4814 Australia; 3 College of Business, Law & Governance, James Cook University, 216/A1 Nguma-bada Campus, PO Box 6811, Cairns, QLD 4870 Australia; 4 Tasmanian School of Business and Economics, University of Tasmania, Melville Street, Hobart, TAS 7001 Australia; 5 Centre for Marine Socioecology, Hobart, TAS Australia; 6 School of Geography, Planning, and Spatial Sciences, University of Tasmania, Grosvenor St, Sandy Bay, Hobart, TAS 7005 Australia

**Keywords:** Angling, Compliance, Marine protected area, Recreational fishing, Theory of planned behaviour

## Abstract

The establishment of marine protected areas with high levels of compliance is a cornerstone of global marine conservation. Previous studies highlight the importance of psycho-social mechanisms (e.g. attitudes, social norms) for driving self-compliance amongst recreational fishers. However, our ability to put these fishery-specific insights into broad-practice requires understanding how transferable they are across locations. In this study, we find that psycho-social determinants of sustainable fishing practices are consistent across three contrasting marine protected areas spanning the Australian continent (separated by up to 3500 km). Intentions to promote sustainable fishing practices were stronger for fishers who were aware of zoning, were positive towards regulations, and whose peers supported sustainable fishing practices. Most of the variability in these factors was linked to intangible fisher characteristics, especially fisher motivations. Consistency across our national sample raises the potential for a national approach to tackling self-compliance of recreational fishers with marine protected area regulations.

**Supplementary Information:**

The online version contains supplementary material available at 10.1007/s13280-025-02227-4.

## Introduction

Under the Kunming–Montreal Global Biodiversity Framework, signatories have committed to effectively protect and conserve 30% of marine areas by 2030 (CBD Secretariat 2022). If achieved, this would be more than a tripling of the current global marine protected area (MPA) estate, with just 8.33% of global oceans in MPAs as of October 2024 (IUCN 2024). For these protected areas to achieve the desired biodiversity outcomes, they must be effectively managed, which includes enforcing regulations and promoting self-compliance with zoning to reduce or eliminate pressures on the ecosystems (Sumaila et al. 2006; Edgar et al. 2014; Johnston et al. 2015; Cinner et al. 2018; Harasti et al. 2019). For many existing or new MPAs, recreational fishers represent important and often dominant marine space users, being present in 76% of the world’s Exclusive Economic Zones (Mora et al. 2009). Achieving compliance in recreational fisheries through enforcement is challenging as fishers are often numerous, widely dispersed and not motivated to comply through enforcement (Cooke et al. 2013; Arlinghaus et al. 2017; Bova et al. 2022). These enforcement challenges are exacerbated by recent trends towards establishing large offshore MPAs where compliance monitoring is cost-prohibitive, potentially compromising intended biodiversity conservation outcomes (Wilhelm et al. 2014). In light of global ambitions for MPAs, finding ways of promoting self-compliance with zoning amongst recreational fishers is a key global biodiversity challenge (Cooke et al. 2013; Edgar et al. 2014).

Seeking self-compliance in a population often requires a deep understanding of that population. Research to date on recreational fishing compliance suggests a range of factors influence pro-environmental behaviour. For example, Thomas et al. (2016) in a New Zealand case study, highlight psycho-social factors, and particularly social norms (community attitudes about what is considered normal and morally acceptable behaviour) to be the strongest determinants of recreational fisher compliance. In contrast, Von Lindern and Mosler (2014) show that fish-stocking behaviours amongst German anglers are influenced by personal attitudes towards fish stocking, and perceived barriers, but not social norms. On the Great Barrier Reef, Bergseth and Rocher (2018) suggest that fishers (miss) perceptions, including inflated perceptions of poaching amongst peers and perceived lack of enforcement, are likely mechanisms for non-compliance. These findings were reinforced by Mackay et al. (2020) in a laboratory setting, showing expectations of others’ behaviours to be the greatest driver of non-compliance behaviours.

**Fig. 1:**
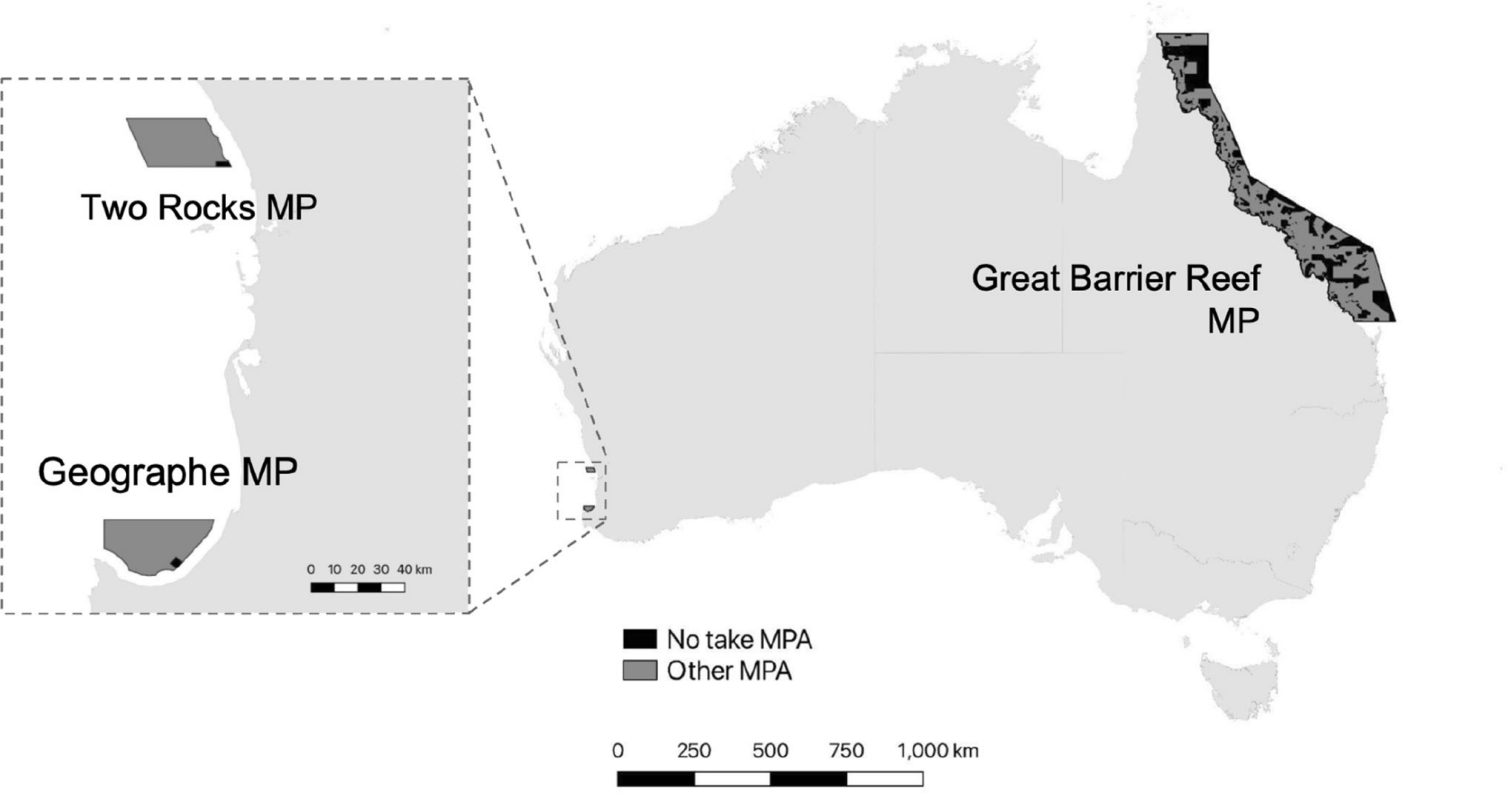
Map showing physical separation of the three case study areas for exploring psycho-social determinants of sustainable fishing behaviours: Great Barrier Reef Marine Park, Geographe Marine Park and Two Rocks Marine Park. These marine parks contain no-take areas (i.e. no extractive activities) alongside areas with more liberal use zoning

These studies highlight the importance of psycho-social mechanisms as determinants of self-compliance amongst recreational fishers. However, our ability to put these fishery-specific insights into broad-practice requires an understanding of how transferable they are across locations. This generalisability is also crucial for a coordinated national or international approach to researching and testing behaviour change interventions for self-compliance in recreational fisheries. In this study, we survey recreational fishers across three contrasting Australian MPAs that span the continent, to examine (i) the consistency of psycho-social determinants influencing recreational fisher behaviour across case studies, and (ii) whether sub-groups of fishers can be targeted for interventions given their individual traits. The three case studies in this study were the Great Barrier Reef Marine Park, Geographe Marine Park and Two Rocks Marine Park (Fig. 1). In Australia, MPAs are commonly referred to as marine parks. These MPAs differ in several key aspects, including physical separation of up to 3500 km, perceived ecological importance (e.g. The Great Barrier Reef is a World Heritage Area (Lucas et al. 1997)), latitude, MPA regulatory structures, MPA age, MPA size, fisher demographics (Moore et al. 2023) and fishery target species (Ryan et al. 2019; Teixeira et al. 2021). These contrasts provide a strong basis for detecting differences in psycho-social determinants of compliance behaviours.

**Fig. 2:**
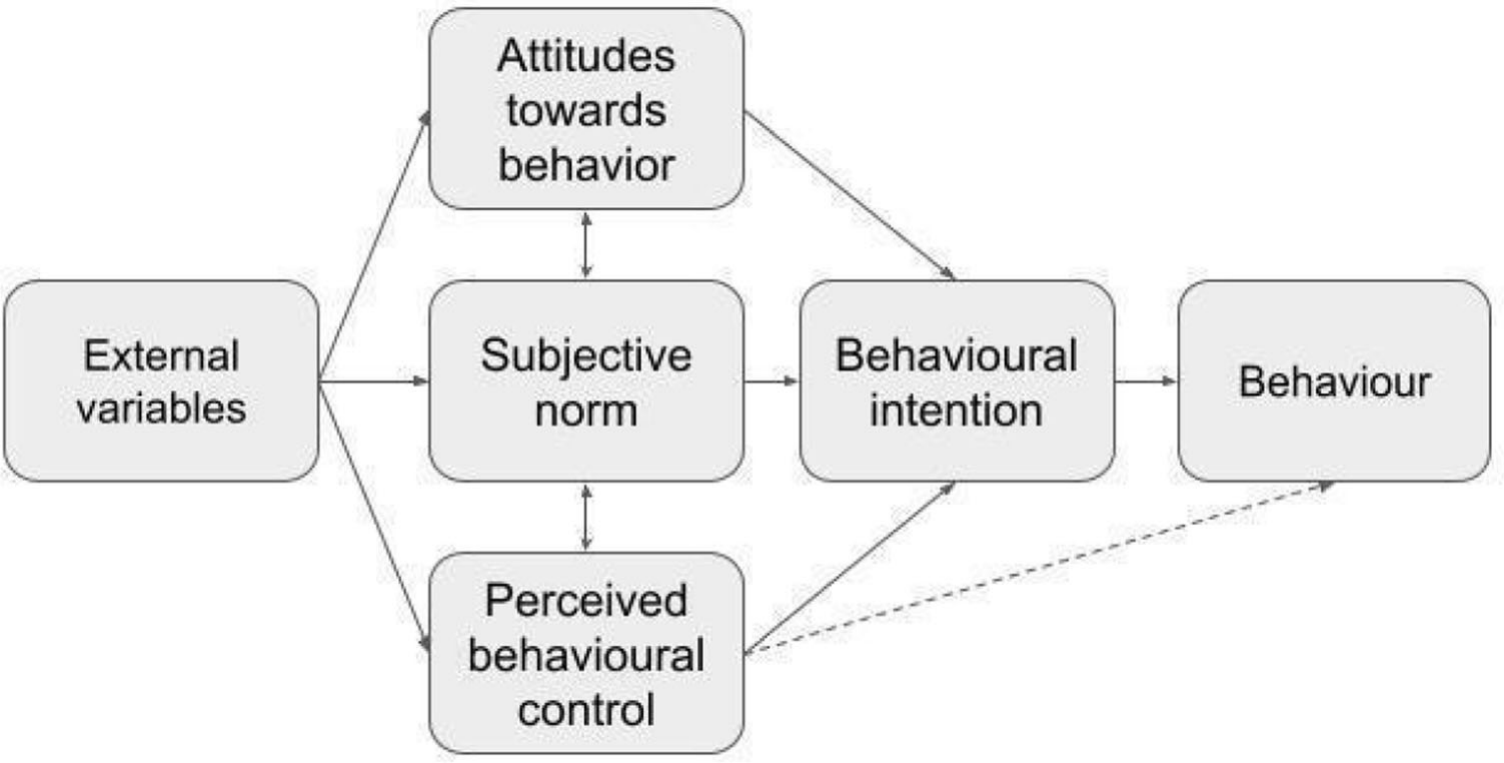
Conceptual diagram of the theory of planned behaviour (Ajzen 1991)

We apply the widely used theory of planned behaviour (TPB) as our primary theoretical model (Ajzen 1991, 2011; Bergseth and Roscher 2018; Bijttebier et al. 2018; Booth et al. 2023), which suggests that behaviours are primarily determined by a behavioural intention. According to the TPB, intention is influenced mostly by three factors: attitudes towards the behaviour, subjective norms (personal understandings of social norms) and perceived behavioural control (perceptions about how easy/hard it is to do the behaviour) (Fig. 2). These constructs are in-turn influenced by external variables, including some externally observable fisher characteristics that might be used to target interventions. Our results show that the drivers of sustainable fishing practices are consistent across our three contrasting MPA case studies, with much of the variability attributable to fisher motivations. We discuss implications for managers seeking to increase self-compliance in MPAs.

## Materials and methods

### Study area

The study considers three case study locations: the Great Barrier Reef Marine Park in Queensland, the Geographe Marine Park in Western Australia and the Two Rocks Marine Park in Western Australia (Fig. 1). The Great Barrier Reef Marine Park covers approximately 344 000 km^2^ across the World Heritage Listed Great Barrier Reef. The current marine park zoning was established in 2014 and includes a 33% allocation to no-take MPAs where all extractive activities (including recreational fishing) are prohibited, alongside more liberal use zoning (Day et al. 2019). Due to the size of the Great Barrier Reef Marine Park, its catchment area includes several urban hubs (e.g. Townsville, Cairns), as well as regional areas. The Great Barrier Reef Marine Park attracts many recreational fishers, including local fishers, and also domestic and international visitors (Teixeira et al. 2021). The smaller Two Rocks Marine Park (882 km^2^), and Geographe Marine Park (977 km^2^), were established in 2018 as part of the Commonwealth managed Australian Marine Parks network. These marine parks are located in offshore waters (starting at three nautical miles from the coastline). Despite their offshore location, both marine parks are regularly visited by recreational fishers (Navarro et al. 2021). Both marine parks include no-take MPAs (approximately 1.5% by area in both marine parks), alongside other more liberal use MPAs (Fig. 1). The Two Rocks Marine Park is adjacent to the Perth Metropolitan Area and is predominantly accessed by local fishers (Navarro et al. 2021; Smallwood and Ryan 2022). In contrast, the Geographe Marine Park is adjacent to the popular tourism towns of Busselton, Dunsborough and Margaret River, attracting a combination of local and visiting fishers (Navarro et al. 2021).

### Questionnaire development

To understand drivers of recreational fisher compliance in these case studies, a questionnaire was developed using the TPB (Fig. 2).

Previous research suggests that direct questioning of recreational fishers about compliance is unlikely to yield accurate responses due to social desirability bias (Tourangeau and Yan 2007; Arias and Sutton 2013). Instead, we opted to use the Net Promoter Score (NPS) as our proxy for behavioural intention, and predictor of actual behaviour in the TPB model. NPS is a 10 point measure of how likely the respondent is to recommend a company, product, service or action. NPS is a widely used construct from the marketing discipline designed to predict actual future behaviour of customers, for example, when measuring brand health, customer loyalty and sales growth (Reichheld 2003). NPS has been widely used as a predictor of behaviour and resulting growth in two thirds of Fortune 1000 companies (Colvin 2020), and across a wide range of fields including healthcare (Adams et al. 2022), hospitality (Agag et al. 2024) and education (Kara et al. 2022).

To apply the NPS in this study, participants were asked how likely they were to recommend sustainable fishing practices to family, friends and other recreational fishers. The responses were recorded on a 10 point scale and classified following the NPS approach into detractors (responses 0 to 6), passives (responses 7 to 8) and promoters (responses 9 to 10). The NPS classifications were used as a proxy for behavioural intention, as recommending sustainable fishing practices provides an indirect indication of whether the individual adopts these practices themselves. No direct questions on actual behaviour were used in this study, as direct-observational data could not be collected to verify if respondents accurately report compliance behaviours.

The survey addressed the main pillars of behaviour according to the TPB. Attitudes were measured by asking fishers a question as to whether they thought leaving areas unfished helps keep fishing sustainable, with responses recorded on a five-point scale. Influence of subjective norms was based on responses to three questions relating to the respondents’ views on whether people around them (including people important to them, people who influence their behaviour and people whose opinions they value) think they should only fish in zones where fishing is allowed. The responses were recorded on five-point scales and reduced using principal component analysis (PCA) to produce a single subjective norm measure. Perceived behavioural control was measured by asking fishers whether they had a good knowledge of the marine park and zones where they fish with responses recorded on a five-point scale.

A wide range of external variables were also measured and included in the analysis. We group these variables into demographic (e.g. age, gender, education etc.), tangible fishing variables (indicating easily observable characteristics of a person’s fishing—e.g. fishing avidity, shore fisher, troll fisher) and intangible fishing variables (indicating hard to observe characteristics of a person’s fishing—e.g. motivations for fishing). A full list of variables, including the external variables used in the analysis, are provided in Table 1.

**Table 1:** Variables used in structural equation model to test the theory of planned behaviour in application to recreational fisher compliance with regulations

Variable	Description
*Behavioural intention*	
NPS classification	Self-reported likelihood of recommending sustainable fishing practices to family, friends and other recreational fishers. Coded as *categorical: Detractor, Neutral, Promoter*
*Attitudes*	
MP attitudes	Self-reported belief that leaving areas unfished helps keep fishing sustainable. Ordinal: *Five-point Likert*
*Social norms*	
Fishing social norms	PCA-reduced score of responses to Likert-items on whether others around the respondent think they should only fish in zones where fishing is allowed. *Continuous*
*Perceived behavioural control*	
MP knowledge	Self-reported knowledge of the marine park zones. Ordinal: *Five-point Likert*
*External: demographic*	
Older fisher (58 years +)	Respondent is over 58 years of age. *Binomial*
Male	Respondent is male. *Binomial*
Local	Respondent lives in a postcode within 50 km of the marine park. *Binomial*
Major city	Lives in a major city of Australia based on Australian Bureau of Statistics remoteness definition. *Binomial*
University degree	Respondent has a university degree or higher level of education (e.g. masters, PhD). *Binomial*
Great barrier reef	Respondent belongs to the Great Barrier Reef sample. *Binomial*
Two rocks	Respondent belongs to the Two Rocks sample. *Binomial*
*External: fishing tangible*	
Avid fisher (15 +)	Respondent is an avid fisher, self-reporting having been fishing more than 15 times in the last 12 months. *Binomial*
Shore fisher	Respondent self-reports mainly fishing from the shore. *Binomial*
Troll fisher	Respondent self-reports troll fishing. *Binomial*
*External: fishing intangible*	
Motivation: be alone/develop skills	PCA score on motivation items most strongly associated with
Motivation: Escape in nature	PCA score on motivation items most strongly associated with
Motivation: Relax and be outdoors	PCA score on motivation items most strongly associated with
Motivation: catch fish to eat	PCA score on motivation items most strongly associated with
Learn from family	Respondent self-reported learning to fish from family members. *Binomial*
Satisfied with catch	PCA score on motivation items most strongly associated with satisfaction with the quantity and quality of fish caught

### Data collection

The survey was distributed using an online paid panel. The survey tool was developed and distributed using Qualtrics survey software. Respondents were first informed that their participation was anonymous and voluntary before consenting to take part in the survey. Screening questions were used to identify respondents who had fished in one of the three case study marine parks (Great Barrier Reef Marine Park, Geographe Marine Park or Two Rocks Marine Park) in the last 12 months. A target sample size of 800 respondents was established, with quotas set at 400 respondents from the Great Barrier Reef case study, 200 from the Two Rocks region and 200 from the Geographe Bay region. These sample sizes meet commonly used thresholds for structural equation modelling (e.g. at least 10 observations per parameter (Bentler and Chou 1987). However, we acknowledge that these thresholds are not reliable (Wolf et al. 2013), and that estimation requirements depend on the nature of the model being estimated (not just the number of parameters). Short of a dedicated Monte-Carlo simulation study, precisely determining sample size requirements is difficult. However, we note that our sample size exceeds all sample size minimums identified in Wolf et al. (2013) using Monte-Carlo simulations for a range of structural equation model configurations.

### Data analysis

Structural equation modelling was conducted using the STATA statistical software. The respondents’ behavioural intention NPS category was modelled using a multinomial logistic regression. Likert-type scale marine park attitudes and marine park knowledge responses were modelled using ordinal regression. The continuous subjective norm PCA scores were modelled using ordinary linear regression. Estimation was performed using full information maximum likelihood.

The structural equation modelling approach allows for investigations into the effect of each element of the TPB on NPS category as well as indirect effects of external variables. These indirect variables affect NPS through attitudes, subjective norms and perceived behavioural control. To help understand the effects of these indirect variables, we decompose their impact on the NPS category by these three mechanisms, obtaining a partial effect (by each of the three pathways) and a total effect. This partitioning of effects was performed using the ‘delta method’ for non linear combinations of parameters (Oehlert 1992). For comparability, effect sizes of continuous variables are scaled to represent the effect of one standard deviation in the independent variable.

## Results

### Are determinants of behaviour shared across regions?

Individuals indicating greater intention to promote sustainable fishing practices (e.g. fish in the right zones and comply with marine park rules) had significantly more positive attitudes about the MPA (MPA attitudes), more positive subjective norms (perception that others around them think they should fish sustainably) and more perceived behavioural control (measured as knowledge of zoning in the MPA, MPA knowledge) (Table 2).

**Table 2:** Logistic regression results from a structural equation model testing the theory of planned behaviour on whether recreational fishers are likely to promote sustainable fishing practices was measured using Net Promoter Score (NPS) categories (Reichheld 2003). The promotion of sustainable fishing practices is positively correlated with marine protected area (MPA) attitudes, social norms and MPA knowledge. NPS-Detractors were used as the base level of the logistic regression. ‘*’ indicates *p* < 0.05, ‘**’ indicates *p* < 0.005, and ‘***’ indicates *p* < 0.001

Variable	Coef	Z	*P*
*NPS-Neutral*			
MPA attitudes	0.44	3.7	< 0.001***
Subjective norms	0.45	3.86	< 0.001***
MPA knowledge	0.27	2.54	0.01*
*NPS-Promoter*			
MPA attitudes	0.99	7.89	0.001**
Subjective norms	0.74	6.19	< 0.001***
MP knowledge	0.35	3.29	0.001**

Interaction effects of MPA attitudes, subjective norms and MPA knowledge with region-specific dummy variables yielded all non-significant effects (Table S1). A likelihood ratio test for the base versus full interaction model failed to reject the base model (X^2^ = 20.05, *p* = 0.06).

### Can interventions be targeted at sub-groups of fishers?

**Fig. 3:**
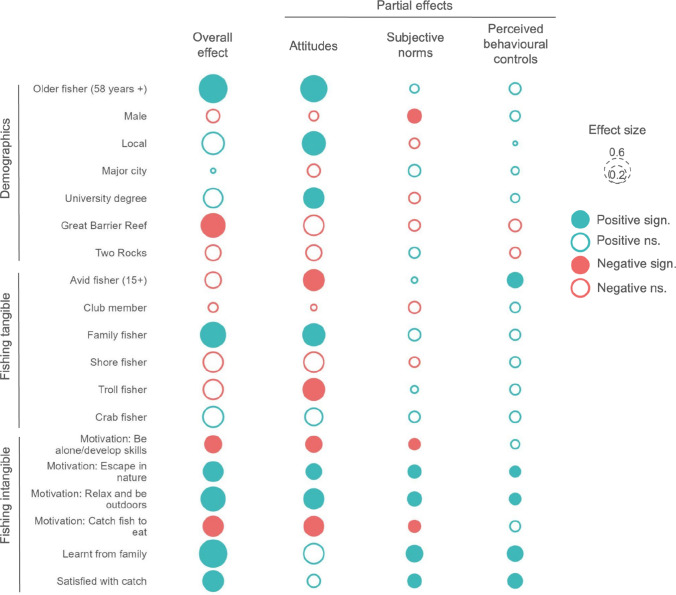
Total and indirect effects of external variables on recreational fishers’ behavioural intentions in relation to promoting sustainable fishing practices in marine protected areas (MPAs). The indirect effects are mediated through measures of the theory of planned behaviour concepts of MPA attitude, subjective norms and MPA knowledge. The total effect reflects the sum of the three mediated effects giving the total effect of the external variable on the behavioural intention. Effect sizes for continuous variables are scaled to one standard deviation

We present the indirect effect of external fisher variables on behavioural intentions as mediated through the TPB measures of MPA attitudes, subjective norms and MPA knowledge. Both the total effect of external variables on behavioural intentions, and partial effects mediated through each of the three TPB measures are included (Fig. 3). Intangible fishing variables were the most influential in terms of effects on MPA attitudes, subjective norms and MPA knowledge, as well as behavioural intentions to promote sustainable fishing practices. In particular, fishers were more likely to promote sustainable fishing practices if they were motivated by a desire to escape in nature, relax and be outdoors, or learnt to fish from family and were satisfied with their catch. These effects were driven by all three TPB pathways, including positive MPA attitudes, subjective norms and MPA knowledge. In contrast, fishers motivated by being alone, practicing skills, or those motivated by catch, were less likely to promote sustainable fishing practices. Most of these effects resulted from negative attitudes towards the MPA. We note that motivations are inherently unobservable, and as such, fishers fishing alone are not necessarily detractors with just 20% of fishers who reported ‘mostly fishing alone’ being detractors.

Demographics and tangible fishing variables tended to have less influence on MPA attitudes, subjective norms and MPA knowledge, as well as the promotion of sustainable fishing practices. Some exceptions were older fishers (over 58 years of age), locals and individuals with university degrees, who tended to have more positive attitudes towards the MPAs, increasing their likelihood of being promoters, although this effect was counteracted for locals and university degree holders by non-significant negative subjective norm effects. Males were less likely to report being influenced by social norms, but counteracting variables meant that males overall were no less likely to be promoters. Respondents from the Great Barrier Reef case study were less likely to be promoters; a result of the cumulative non-significant negative effect of MPA attitudes, subjective norms and MPA knowledge. Tangible fishing variables had no significant indirect effect, whilst avid fishers tended to have higher MPA knowledge they were not more likely to be promoters of sustainable fishing practices.

## Discussion

Previous research has shown psycho-social factors are important as determinants of recreational fishers’ compliance with MPAs and adoption of sustainable fishing practices (Thomas et al. 2016; Bergseth and Rocher 2018; Mackay et al. 2020). In this paper, we show commonality in the effects of psycho-social factors on sustainable fishing practices across three contrasting MPAs spanning the Australian continent. The implication of this finding is in support of a national, and potentially international approach, to tackling the challenge of self-compliance of recreational fishers with MPA regulations. Our research also shows non-tangible fisher characteristics, particularly fisher motivations, are important for understanding sustainable fishing practices amongst recreational fishers.

### A national approach to self-compliance

Fishers across our three case studies had similar drivers of behavioural intentions, which raises the potential for national scale efforts to tackle self-compliance. Whether TPB effects are expected to be generalisable or context specific is an unresolved issue that has received limited attention. Hassan et al. (2016) conducted a systematic review of cross-country TPB studies on green purchase decision, finding consistent effects of attitudes and perceived behavioural control, but differing effects of subjective norms. They provide some evidence that the differences in subjective norm effects are associated with cultural dimensions such as power distance. Whilst the three MPAs included in our study are contrasting in terms of geography, size, ecology and age, the recreational fishers that use them are likely to share common fundamental cultural norms and beliefs (at least on average) and as such commonality in drivers of behaviours is perhaps not surprising, although no data were collected on cultural norms and beliefs in our survey. Further research is needed to confirm whether fishers from different countries and socio-cultural contexts share psycho-social determinants of compliance behaviours. Nevertheless, this study supports the idea of shared psycho-social determinants across at least the three case studies examined here spanning the Australian continent (with the Western Australian and Queensland study sites separated by approximately 3500 km).

In practice what is meant by a ‘national approach to self-compliance’ remains an open question. Most obviously, our findings suggest that interventions effective in one location are likely to work in others. As such, managers across jurisdictions should cooperate, share insights and form a national-level knowledge base of effectiveness of different interventions. There are several changes in practices that are required to help build this knowledge base. Firstly, whilst compliance represents a major challenge to the conservation effectiveness of MPAs (Cooke et al. 2013; Edgar et al. 2014), relatively little data are available on compliance rates in recreational fisheries, and by extension the effectiveness of strategies to increase compliance (Bova et al. 2022). Generating a knowledge base of intervention effectiveness requires robust and (to the extent possible) consistent monitoring approaches. Secondly, as outlined by van Valkengoed et al. (2022), to understand why a compliance intervention worked or not requires reflection on which behavioural determinants were targeted by the intervention and if those changed. As such, monitoring should not be limited to the behavioural outcome itself, but also targeted behavioural determinants.

### Designing and targeting interventions to address non-compliance

Compliance strategies for MPA within Australia (and we suspect in other jurisdictions around the world) have largely focussed on deterrence through enforcement and providing information (e.g. zoning maps at boat ramps) (Bergseth 2017). Deterrence through enforcement is necessary, but not sufficient for addressing compliance challenges in MPAs (Arias 2015). Even with high compliance efforts in the Great Barrier Reef Marine Park, recreational fishers perceived likelihoods of getting caught if poaching is low (Arias 2015). Alternatively, information provision as a strategy for promoting compliance relies on the largely discredited assumption that the cause of non-compliance is a lack of information (Kahan et al. 2012; Bergseth 2017). As discussed by Bergseth (2017), new behaviour change strategies are needed to address the compliance challenges of MPAs.

Our study finding that attitudes, subjective norms and perceived behavioural control all have significant effects on intentions to promote sustainable fishing practices raises possibilities for promoting self-compliance. One option suggested by Bergseth and Roscher (2018) is to use messaging to communicate high compliance rates and a norm of compliance amongst recreational fishers, correcting for the over-inflated perceived rates of poaching found in their study. Inversely, avoiding communications about non-compliance events including social media posts when people have been fined (a common practice in many fisheries agencies across the world) could also help address the over-inflated perception of poaching and reinforce a norm of compliance (Cialdini 2003; Thomas et al. 2016). Alternatively, making it ‘easy’ to comply with regulations could be an effective strategy, by increasing perceptions of behavioural control. This could be achieved by developing navigational tools for zoning, with features to provide an alert when a fisher enters a no-take MPA. In terms of attitudes, research showing that recreational fishers’ support of MPAs increases over time suggests compliance may also be expected to increase (Navarro et al. 2018). Combinations of approaches that are effectively targeted at sub-populations are likely to be most effective (Michie et al. 2011).

To inform targeting of approaches, our analysis of the effect of external variables is useful. Our results show that much of the variation in attitudes, subjective norms and perceived behavioural control was explained by non-tangible fishing related variables and particular motivations for fishing. Motivations for fishing have been relatively well explored as drivers of attitudes to MPAs (Voyer et al. 2014; McNeill et al. 2019), but rarely in the context of compliance (Arias and Sutton 2013). The importance of non-tangible factors presents a challenge for targeting interventions. For example, while it is relatively easy to direct social marketing campaigns at specific age groups, targeting fishers based on their motivations is more complex. Further research is needed to identify effective ways to reach fishers with specific motivations. However, once engaged, various strategies can be used to design campaigns that resonate with their motivations. For example, fishers motivated to be alone and to catch fish were less likely to promote sustainable fishing practices, with this largely attributable to negative attitudes towards regulations (noting that being motivated to fish alone is different to actually fishing alone, and as such compliance efforts cannot simply target lone fishers). Given the likely challenge of shifting attitudes amongst detractors, one prudent approach might be to instead focus on removing obstacles to compliance for these fishers–perhaps by developing tools such as fishing apps to help anglers navigate MPAs (Cooke et al. 2021). Pairing zoning information with safety features such as hazard navigation (important for solitary fishers) and information to help maximise catch (important for catch motivated fishers) may help increase the appeal of these tools.

### Study limitations

The current study has several limitations that should be considered in interpreting the results. Our use of an online paid panel may under-represent some segments of the populations (e.g. individuals with limited computer and internet access) (Lehdonvirta et al. 2020). Additionally, research on compliance behaviours inevitably face measurement challenges, with direct questioning on compliance unlikely to yield reliable responses (Tourangeau and Yan 2007; Arias and Sutton 2013). In this study, we addressed the issues of direct reporting by using an NPS measure of how likely respondents are to recommend sustainable fishing practices. Whilst this strategy potentially minimises social desirability bias, individuals may report recommending sustainable fishing practices even if they do not practice those behaviours themselves. More broadly, as modelling of psycho-social determinants occurs at the individual level, novel survey techniques focussed on overcoming social desirability bias for accurate population-level reporting (e.g. random response technique) are not suitable (Arias and Sutton 2013). As such, to improve modelling of the psycho-social determinants of compliance behaviours, there is a need to develop and test robust approaches for measuring non-compliance behaviours at the individual level.

## Conclusions

The importance of recreational fishing in many countries highlights the need for increased efforts in enhancing voluntary compliance with MPA regulations. Applying the TPB to study psycho-social determinants of sustainable fishing practices, our results suggest that managers have a wide suite of under-utilised behaviour change tools at their disposal to promote voluntary compliance amongst recreational fishers. These strategies could target attitudes towards MPAs, subjective norms or perceived behavioural controls. Specific examples include establishing a subjective norm through campaigns to communicate high compliance rates amongst recreational fishers, or making compliance easier using zoning apps that notify recreational fishers when they enter a no-take MPA. Our results show that to maximise the impact of these strategies managers should tailor campaign design to target fishers based on their motivations for fishing. In particular, fishers motivated to be alone and to catch fish for food were less likely to report promoting sustainable fishing practices, suggesting campaigns targeted to appeal to these fishers may be particularly effective in increasing voluntary compliance.

Despite being separated by up to 3500 kms, we also found that psycho-social determinants of sustainable fishing practices amongst recreational fishers were consistent across three contrasting Australian MPAs. The consistency in our cross-continental study of recreational fisher compliance provides support for national, and potentially international, collaborative efforts to improve voluntary compliance amongst recreational fishers. We propose that this collaboration could be facilitated by adopting consistent intervention effectiveness monitoring practices, ensuring that monitoring captures effects on both the targeted behaviour and its psycho-social determinants, and that information is compiled in national or international knowledge bases to ensure effective knowledge sharing. Doing so would help managers identify and scale-up use of the most effective strategies. This scaling would form a critical complement to traditional compliance strategies (e.g. enforcement) in ensuring that MPAs can achieve their intended biodiversity conservation objectives.

## Supplementary Information

Below is the link to the electronic supplementary material.

Supplementary file1 (DOCX 20 KB)

## Data Availability

The data that support the findings of this study are available from the corresponding author, but restrictions apply to the availability of these data, which were collected under a human ethics agreement with stipulated data use cases. Please contact the corresponding author to discuss data access.
