# Supplementary material for: Hooking recreational fishers on sustainable fishing: Consistent psycho-social determinants raise potential for broad scale interventions
Source: Ambio. 2025 Aug 21;55(2):360–70. doi: 10.1007/s13280-025-02227-4 (PMC12779826; doi:10.1007/s13280-025-02227-4)
Supplement: Supplementary file 1 — Supplementary file1 (DOCX 20 KB) [file 13280_2025_2227_MOESM1_ESM.pdf]

Ambio

Supplementary Information

This supplementary information has not been peer reviewed.

Title: Hooking recreational fishers on sustainable fishing: consistent psycho-social determinants raises potential for broad scale interventions

Table S1: Logistic regression results from a structural equation model testing the Theory of Planned Behaviour in relation to whether recreational fishers are likely to promote sustainable fishing practices, with full case study interactions.

Variable	Coef.	Z	P
NPS-Neutral			
MPA Attitudes: GBR MP	0.39	2.89	0.004**
MPA Attitudes: Two Rocks MP	0.60	3.40	0.001**
MPA Attitudes: Geographe MP	0.44	2.33	0.020*
Subjective Norms: GBR MP	0.58	3.77	<0.001***
Subjective Norms: Two Rocks MP	0.09	0.36	0.721
Subjective Norms: Geographe MP	0.400	1.57	0.116
MPA Knowledge: GBR MP	0.298	2.27	0.023*
MPA Knowledge: Two Rocks MP	0.031	0.17	0.864
MPA Knowledge: Geographe MP	0.453	2.27	0.023*
NPS-Promoter			
MPA Attitudes: GBR MP	0.96	6.76	<0.001***
MPA Attitudes: Two Rocks MP	1.13	6.27	<0.001***
MPA Attitudes: Geographe MP	0.98	5.05	<0.001***
Subjective Norms: GBR MP	0.88	5.59	<0.001***
Subjective Norms: Two Rocks MP	0.33	1.35	0.176
Subjective Norms: Geographe MP	0.77	2.77	0.006**
MPA Knowledge: GBR MP	0.44	3.36	0.001**
MPA Knowledge: Two Rocks MP	0.13	0.72	0.472
MPA Knowledge: Geographe MP	0.44	2.15	0.032*

Table S2: Principal component analysis using orthogonal varimax Kaiser rotation, to predict values for components motivations for fishing survey items. Predicted 5 components, Rho = 0.6496. Scoring coefficients from PCA analysis set out in table below.

Survey item	Comp 1: Escape in nature	Comp 2: Be alone/ develop skills	Comp 3: Relax & be outdoors	Comp 4: Catch fish to eat	Comp 5: With family & friends
Relaxing/unwinding	0.03	-0.02	0.63	0.02	0.04
Spending time in nature	0.03	0.04	0.61	-0.02	0.05
Spending time on my own	-0.02	0.50	0.31	-0.13	-0.12
Spending time with family	-0.04	-0.02	0.08	-0.06	0.71
Spending time with friends	0.02	-0.02	0.01	0.07	0.66
Competing in fishing competitions	-0.10	0.58	-0.11	0.11	0.04
Experiencing physical and mental challenges due to weather, terrain, or the process of catching fish	0.02	0.50	0.03	0.07	-0.02
Catching fresh fish for myself and my family to eat	0.01	-0.03	0.08	0.73	-0.04
Catching fresh fish to give to friends and others	0.05	0.14	-0.10	0.59	0.06
Catching fish to release	0.29	0.34	-0.25	-0.25	0.12
Being able to focus on fishing and not think about other things	0.46	-0.11	0.13	0.05	-0.13
Learning about nature / the environment	0.51	-0.05	0.10	0.00	-0.06
Having conversations with others about topics I wouldn’t usually discuss	0.50	-0.02	-0.12	0.05	0.09
Getting exercise	0.42	0.09	-0.02	-0.03	0.05
Proportion of variance explained by component	*0.1754*	*0.1358*	*0.1196*	*0.1114*	*0.1074*

Table S3: Principal component analysis using orthogonal varimax Kaiser rotation, to predict values for components motivations satisfaction items. Predicted 2 components, Rho = 0.6496. Scoring coefficients from PCA analysis set out in table below.

Survey item	Satisfaction with catch	Satisfaction with management
How satisfied are you with the quantity of fish caught during your saltwater fishing	0.74	-0.07
How satisfied are you with the quality of fish caught during your saltwater fishing	0.67	0.08
How satisfied are you with the environmental management of the saltwaters you fished	0.01	0.99
Proportion of variance explained by component	*0.5624*	*0.3335*

Table S4: Principal component analysis using orthogonal varimax Kaiser rotation, to predict values for components subjective norm items. Predicted 2 components, Rho = 0.8511. Scoring coefficients from PCA analysis set out in table below.

Survey item	Subjective norms	Website information
People who are important to me think I should only fish in the marine zones where fishing is allowed	0.54	0.10
People who influence my behaviour think I should only fish in the marine zones where fishing is allowed	0.60	-0.03
People whose opinions I value prefer that I only fish in the marine zones where fishing is allowed	0.60	-0.07
Website information has been helpful for me to know where I am allowed to fish, and where there are restrictions on fishing	0.00	0.99
Proportion of variance explained by component	*0.5988*	*0.2523*
